# Virulence-associated type III secretion systems in Gram-negative bacteria

**DOI:** 10.1099/mic.0.001328

**Published:** 2023-06-13

**Authors:** Sara Vilela Pais, Eunjin Kim, Samuel Wagner

**Affiliations:** ^1^​ Interfaculty Institute of Microbiology and Infection Medicine (IMIT), University of Tübingen, Tübingen, Germany; ^2^​ Excellence Cluster "Controlling Microbes to Fight Infections" (CMFI), Tübingen, Germany; ^3^​ Partner-site Tübingen, German Center for Infection Research (DZIF), Tübingen, Germany

**Keywords:** bacterial pathogenicity, type III secretion systems

## Abstract

Virulence-associated bacterial type III secretion systems are multiprotein molecular machines that promote the pathogenicity of bacteria towards eukaryotic host cells. These machines form needle-like structures, named injectisomes, that span both bacterial and host membranes, forming a direct conduit for the delivery of bacterial proteins into host cells. Once within the host, these bacterial effector proteins are capable of manipulating a multitude of host cell functions. In recent years, the knowledge of assembly, structure and function of these machines has grown substantially and is presented and discussed in this review.

## Introduction

**Fig. 1.**
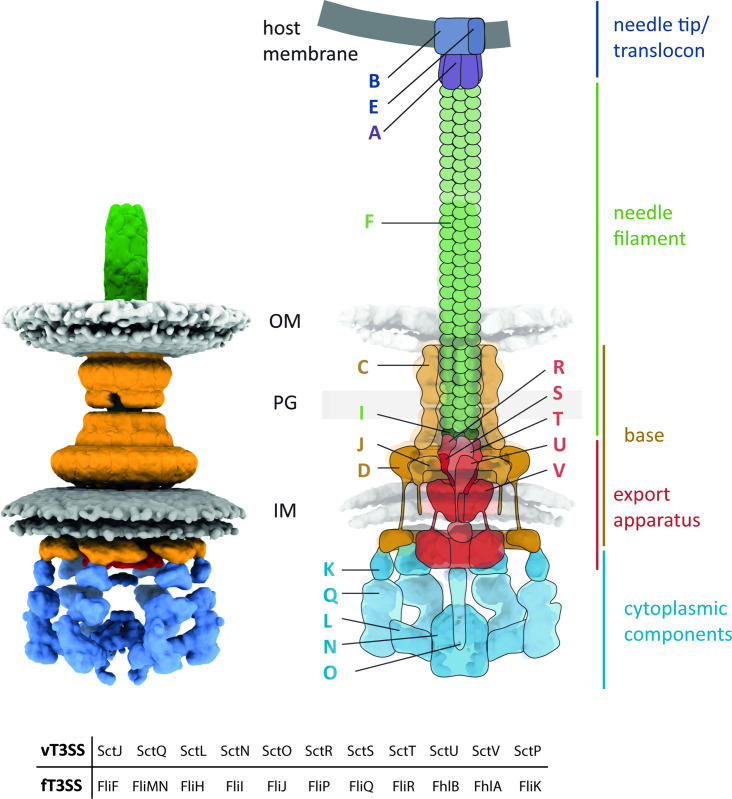
Structure and components of a type III secretion system (T3SS) injectisome. *Left:* Subtomogram of T3SS-1 injectisome of *Salmonella enterica* serovar Typhimurium, (accession code EMD-8544) [35]. *Right:* Overlay of sliced-tomogram with a model of T3SS depicting all the individual components of the injectisome. *Below*: Table showing conserved proteins between virulence T3SS (vT3SS) and flagellar T3SS (fT3SS). Protein names follow the unified nomenclature according to Wagner and Diepold [155] and are depicted as letters (e.g. SctO: O). The different structural units are colour-coded. Adapted from Wagner [102] . IM: inner membrane, PG: peptidoglycan, OM: outer membrane.

Virulence-associated-type III secretion systems (T3SS) utilize a supramolecular protein complex that is also termed injectisome for secretion of bacterial effector proteins into eukaryotic host cells. The injectisome evolved from the flagellar T3SS that serves bacterial motility, and a high level of structural similarity exists between the proteins that constitute the injectisome and those that constitute the flagella [1]. The injectisome has a molecular mass of around 7 MDa and is composed of more than 200 subunits of up to 20 different proteins [2]. It spans the inner (IM) and outer (OM) membranes of Gram-negative bacteria as well as a membrane of the host. It is built up of a base, an export apparatus, cytosolic components including an ATPase and a needle filament including a needle adapter and a needle tip (Fig. 1). T3SS adapted to the needs of different pathogens and as a result, several families of T3SS can be found [3]. While the overall structure of the injectisome and the export mechanism are conserved, the components of T3SS that directly contact the host, like the needle tip and translocon, are very different between bacterial species [4]. Also, the regulatory mechanisms of gene expression, assembly and secretion as well as the secreted effectors vary among bacteria and even in the same bacteria (i.e. *Salmonella *’s T3SS-1 and T3SS-2) depending on pathogen needs.

Much new structural and functional information on the injectisome has been produced in recent years, which has led to a substantial elucidation of the organization of this machinery, of the understanding of the dynamic mechanisms that drive injectisome assembly, and of the targeting and passage of substrates through the injectisome channel. In this review, we summarize and discuss these recent findings of the field, starting with the structural components.

## Export apparatus

The export apparatus is composed of five highly conserved proteins, SctR, SctS, SctT, SctU and SctV, with a stoichiometry of 5 : 4 : 1 : 1 : 9 [2, 5–7] (where proteins are equivalent, we also refer to publications on the bacterial flagellum). The minor export apparatus proteins SctR, SctS and SctT contain four, two, and six predicted transmembrane segments (TMS), respectively, and together form a unique helical structure called the core export apparatus [5, 8]. One SctR and one SctS are structurally equivalent to SctT, so that the SctR_5_S_4_T_1_ complex is of pseudo-hexameric symmetry [5, 8]. Despite its substantial hydrophobicity, the SctR_5_S_4_T_1_ complex is not integrated in the inner bacterial membrane in the final structure but it is located on the periplasmic side at the centre of the base with hydrophobic contacts to a nonameric ring of the transmembrane domains of SctV [5, 9, 10].

As the core export apparatus forms a pore in the IM, it is crucial that the pore is gated and opens only during substrate translocation. The export apparatus has several constriction points that undergo conformational changes upon substrate engagement [11, 12]. There are hydrophilic Q1 and Q2 belts that sandwich a hydrophobic M-gate [11, 12]. The substrate is first engaged by the Q1 belt, which is formed by glutamic acid residues from the four SctS subunits. Once stably engaged by the Q1 belt, the substrate is engaged by the M-gate, which is formed by conserved methionines in the SctR proteins. Subsequent movement of the flexible SctT plug opens the upper atrium of the export apparatus where is located the Q2 belt, formed by SctR subunits. The Q2 belt further engages the substrate during translocation. These constriction points are narrow and substrates must be unfolded to go through the passage; the narrowest is the M-gate, which measures~5.5 Å in diameter [6, 12]. Several conserved inter- and intramolecular salt bridges are present in the export apparatus, which have been shown to be important for assembly and stabilization of the complex [13], and some are also involved in gating of the export apparatus [5, 11–13].

The switch protein SctU contains four predicted TMS and a cytoplasmic domain. These TMS form two α-helical hairpins that resemble the structure of SctS, making SctU the closing component of the SctR_5_S_4_T_1_ complex [6]. SctU contains a highly conserved loop between the two hairpins that wraps around the entrance of the export gate [6]. Although the specific role of the loop is unclear, the interaction of the loop with each SctS subunit has been shown to be important for secretion function and the loop likely plays a role in the gating mechanism [6]. The cytoplasmic domain of SctU contains a highly conserved NPTH motif, which undergoes autocleavage [14–16]. It has been proposed that the autocleavage is the trigger for the substrate switching [17, 18] but it is possible that the autocleavage causes conformational changes in SctU that are required for its function in substrate specificity switching [19].

The major export apparatus protein SctV forms a sea-horse like structure as shown by cryo-electron tomography (cryo-ET) and contains a transmembrane domain (TMD) with eight predicted TMS, a cytoplasmic domain, and a linker that connects the two domains [9]. The atomic structure of the TMD of SctV has not been determined yet but the cryo-ET structure revealed that the TMDs of SctV wrap around the lower part of the export apparatus through hydrophobic interactions [9]. The cytoplasmic domain forms a nonameric ring with a pore size of 50 Å [9, 20–22]. It is postulated to serve as a docking platform for substrates and chaperones and to be involved in the specificity switching of secretion [9, 23, 24].

## Base

The base is composed of the IM and OM rings. SctD and SctJ are components of the IM ring, where a SctD 24-mer ring (~250 Å wide) encompasses a smaller SctJ 24-mer ring (~180 Å wide) [25, 26]. SctD is anchored in the IM with a TMS located between three periplasmic domains and a cytoplasmic domain [25]. The cytoplasmic domain of SctD functions as a platform for interaction with the cytoplasmic components of the injectisome. The three periplasmic domains each contain topologically similar ring building motifs (RBMs) [27]. The RBM is composed of two α-helices folded against a β-sheet. It is present in all proteins that build the base structure (SctCDJ) and aids in the formation of the ring [27].

The smaller IM ring protein SctJ is anchored to the IM by an N-terminal lipid anchor and in addition by a C-terminal TMS in some T3SS [25, 28]. The two periplasmic domains of SctJ contain RBMs that constitute the concentric ring architecture of SctJ and provide inter-subunit interactions [27, 28]. The SctJ ring clamps the core export apparatus and supposedly brings it into its juxtamembrane position during assembly [5].

Above the IM rings, SctC comprises the OM ring. SctC contains three N-terminal domains (N0, N1 and N3), a secretin domain, and an S domain. The N-terminal domains contain the modular RBM [27], which aid in the formation of the SctC ring [26]. N0 and N1 domains constitute the ring below the periplasmic constriction and the N0 domain connects the OM ring to the IM ring of SctD. The N3 domain, together with the secretin and S domains, forms a pore structure above the periplasmic gate [29]. The secretin domain displays a double-walled β-barrel structure. In the lumen of the β-barrel, the inner wall acts as a transient periplasmic gate (~15 Å wide) to block access from the extracellular space, before needle filament polymerization opens up the gate [10, 29, 30]. The S domain is located outside of the barrel and functions as a binding site for pilotin SctG, a chaperone that is involved in SctC targeting and assembly in the OM [31, 32]. The outer wall of the secretin contains an amphipathic loop and hairpin at the distal end, which anchor the SctC ring to the OM [29]. SctC has repeatedly been shown to form a 15-mer concentric ring, which seemed unconventional to assemble with the 24 subunits of the SctD IM ring (ratio of 5 : 8) [25, 26, 29]. It has been revealed recently that the SctC ring possesses a sixteenth SctC molecule that only contains the N0 and N1 domains. This accessory domain resolves the symmetry mismatch between SctC and SctD in such a way that always two adjacent C-terminal domains of SctD interact with two N-terminal domains of SctC. The C-terminal domain of each third SctD does not participate in this interaction and folds somewhat to the outside of the base [10, 33, 34]. Interestingly, this partial SctC molecule only exists in the presence of the export apparatus. The absence of the latter results in a 15 : 23 stoichiometry between the OM and IM rings [34], showing how critical a precisely orchestrated assembly is for a functional injectisome. However, it is unclear at this point when the sixteenth SctC molecule is recruited and processed to its final form.

## Cytoplasmic components

**Fig. 2.**
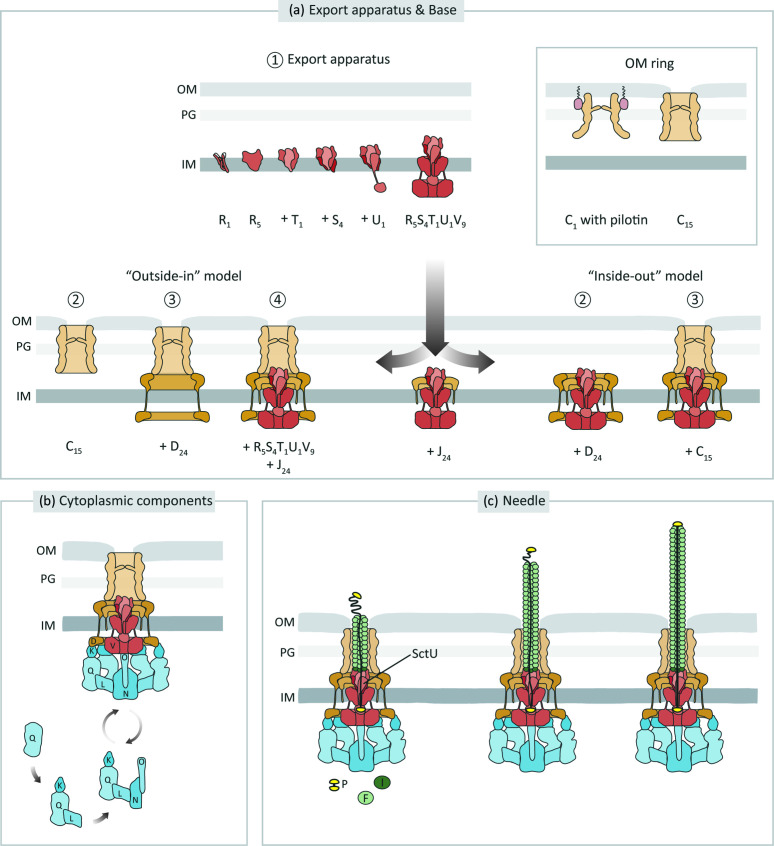

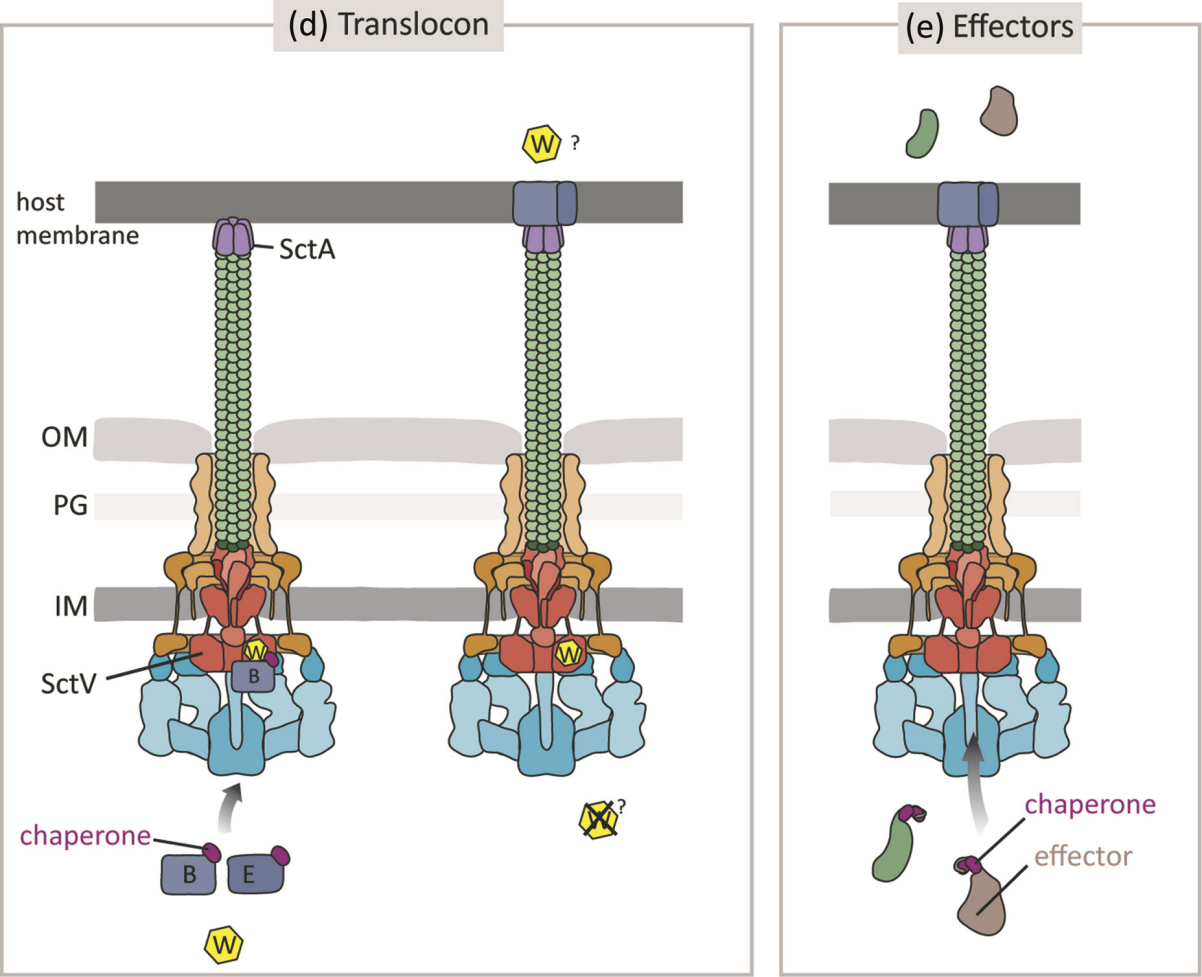
Assembly of the type III secretion system. (**a**) Assembly of T3SS begins with formation of the export apparatus and the membrane rings, which follows either ‘inside-out’ or ‘outside-in’ models. In both models, the export apparatus self-sufficiently assembles and the smaller IM ring (SctJ) forms around the export apparatus. In the inside-out model, it is followed by the formations of the bigger IM ring (SctD) and OM secretin ring (SctC), in that order. SctC is recruited and oligomerized in the OM often with help of pilotin. In the outside-in model, the SctC ring formation is required for the SctD ring assembly. The export apparatus with the SctJ ring is then incorporated into the SctCD rings. (**b**) The sorting platform assembly and recruitment to the export apparatus and base is a dynamic and cyclic process. The sorting platform is capable of interacting with T3SS chaperone-substrate. (**c**) The assembly of the needle involves the secretion of early substrates SctI, the needle adaptor, followed by secretion of SctF, the needle filament. Needle length is regulated by SctP, which further interaction with SctU leads to switch of secretion to the intermediate substrates. (**d-e**) Translocators (SctBE) are then secreted and inserted into the host membrane where they form a pore. The needle tip guides translocon insertion. SctW regulates intermediate substrate secretion, by binding to SctV. Once SctW is degraded or secreted, switch to secretion of late substrates occurs and effectors are deployed into the host cells. Protein names follow the unified nomenclature according to Wagner and Diepold [155] and are depicted as letters (e.g. SctO: O). The different structural units are colour coded. Adapted from Wagner [102]. IM: inner membrane, PG: peptidoglycan, OM: outer membrane.

The cytoplasmic components of the injectisome comprise the proteins SctQ, SctL, SctO, SctN and SctK (Fig. 2b). The structural analysis of these components has been hampered by the difficulty of isolating this structure, possibly due to its dynamic nature. Cryo-ET analysis revealed that the cytoplasmic components form a cage of six pod-like structures comprising SctK and SctQ. These emerge from the needle complex towards the cytoplasmic side, forming a scaffold for a centrally positioned ATPase (SctN) that connects to the pods through six spoke-like structures of SctL. The central ATPase is connected to SctV by one molecule of SctO [35, 36].

The pods connect to the cytoplasmic domain of the base inner ring protein SctD through SctK [35, 37]. SctK is composed of nine α-helices and folds into a kidney-like structure [38]. In addition to its base-associated form, SctK was also shown to form soluble complexes with the other cytosolic components [39–41]. The other pod component, SctQ, contains an internal translation start site, resulting in the expression of both full-length (SctQ_FL_) and C-terminal fragment (SctQ_C_) [42–44]. SctQ_FL_ and SctQ_C_ interact to form heterotrimers in a stoichiometry 1 SctQ_FL _: 2 SctQ_C_ [45], where SctQ_C_ binds to the N-terminal domain of SctQ_FL_. This interaction probably stabilizes SctQ_FL_ which is unstable without SctQ_C_ [42, 45–47]. SctQ_FL_ and SctQ_C_ are essential for function but the exact role of SctQ_C_ is still controversially discussed [45, 47].

SctL connects the pods in a spoke-like fashion to the ATPases SctN, forming a hub. The N-terminus of SctL interacts with SctQ_FL_ [35, 45, 48], while its C-terminus interacts with the N-terminus of SctN [45]. *In vitro*, SctL is insoluble by itself but forms soluble, stable complexes of SctQ_FL_/SctQ_C_/SctL, SctQ_FL_/SctQ_C_/SctL/SctN and SctL/SctN [45].

The ATPase SctN shares structural homology with the F1-ATPases and forms a hexameric ring containing six catalytic sites in different states [49], consistent with the rotary mechanism adopted by the F_1_-ATPases. Each SctN monomer comprises three subdomains. First, a C-terminal domain containing five α-helices forms the surface of the SctN pore. Then, a central ATPase, essential for catalytic activity, contains mixed A/B Rossman fold and Walker A and B motifs, typical of ATPases. Lastly, a N-terminal domain is required for oligomerization. The ATPase ring complex is similar to the cytoplasmic part of the F_o_F_1_ ATP synthases, where a rotary motor couples proton flow through F_o_ with ATP synthesis by F_1_. SctN was shown to dissociate the chaperone-substrate complexes by hydrolysis of ATP and to unfold substrates [50]. However, it is difficult to integrate this observation into a model where substrate-chaperone complexes also bind to the cytoplasmic ring of SctV.

SctO forms helical coiled-coil hairpins like its flagella counterpart FliJ [36, 49, 51–53]. The latter is structurally similar to the central stalk proteins of rotary ATPases. SctO links SctN and SctV. On one side, the N- and C-termini of SctO locate to the SctN C-terminal pore, where a SctN negatively charged region adjoins a positively charged region of SctO [49]. On the other side, SctO binds to a cleft between two different SctV protomers [51]. This interaction occurs in a region of SctV also described to bind chaperone-substrate complexes [54].

## Needle structure

The needle filament is a helical conduit emerging from the export apparatus. It is anchored to the export apparatus by six molecules of the needle adapter protein SctI (also known as inner rod) [2, 7, 10, 12, 55]. SctI forms an alpha helical hairpin with the termini facing towards the core export apparatus and it is able to adapt to different structures, depending on the position. SctI_1_ binds extensively to the interface between SctR and SctT, while being partially ordered. The SctI_2-6_ molecules display more ordered helices and interact at the SctR-R and SctR-T interfaces, as well as with the surrounding SctC structure [10, 12]. The ability to adopt different structural conformations and establish different interactions allows SctI to function as a needle adaptor providing a homogenous platform for the following needle filament assembly [10, 12]. The needle filament is a stiff rod composed of SctF protomers that are structurally similar to SctI [56]. The C-terminal half of SctF faces the lumen of the needle filament. It measures about 15 Å in diameter, exhibits a right-handed spiral shape and is lined with conserved charged amino acid residues that are critical for the substrate secretion process [30, 57]. The N-terminus of SctF extends to the outside of the filament and lacks secondary structure [30]. The needle filament measures around 50 nm in length and its extent varies between bacterial species [58–60].

## Needle tip

At the distal end of the needle filament, SctA assembles a needle tip complex, which is mostly found to be pentameric [61–63]. A recent structural study on the *Salmonella* needle tip complex has revealed that the complex forms a continuous conduit from the needle filament (~80 Å wide and ~800 Å long) but with a narrower opening at the distal end (~10 Å) [62]. The SctA structure is composed of a central coiled-coil domain in between the N- and C-terminal α/β domains [61, 64–67]. The coiled-coil domain is a conserved structural component across the T3SS needle tip homologs. It is involved in the interaction with the needle filament and oligomerization, and makes up the lumen of the tip complex [61, 64, 68]. Interestingly, the surface electrostatic properties of the lumen of the tip complex and of the needle complex are distinct, as the lumen of the tip complex is predominantly negatively charged [62]. However, the physiological relevance of this difference remains unclear. On the other hand, the N-terminal domain seems to differ in the structure depending on the bacterial species and the presence of a cognate chaperone. In *Salmonella *, *Shigella *, and *Burkholderia *, the N-terminal domain exhibits an α-helical hairpin structure and has been shown to act as a self-chaperone to inhibit premature oligomerization in the bacterial cytosol by binding to the C-terminal coiled-coil domain [64]. In *Yersinia* and *Pseudomonas *, the N-terminus of SctA has a globular domain and a separate cognate chaperone is required to prevent premature oligomerization and for successful secretion of SctA [63, 68–71]. In EPEC and EHEC, SctA does not form a pentameric complex but extends as a unique filamentous structure on top of the needle [72–74]. The lumen of the SctA filament has a similar diameter as the one of the needle filament and also similar electrostatic properties featuring a spiral hydrophobic groove lined with positively charged residues [75, 76]. The SctA homolog of EPEC and EHEC mainly consists of a conserved coiled-coil domain with a small, kinked N-terminal helix [28, 75, 76]. In addition, SctA in EPEC and EHEC also requires a cognate chaperone to inhibit premature polymerization [28, 77].

## Translocators

The hydrophobic translocator proteins SctB and SctE form the translocon pore in the host cell membrane. SctB is the major and SctE is the minor translocator and both share a dedicated chaperone (see *Targeting and transport across the injectisome*). The major and minor translocators are predicted to contain two and one transmembrane helices, respectively. Additionally, these proteins contain coiled-coils and intrinsically disordered regions. Due to their nature, only the structures of translocators interacting with their respective T3SS chaperones have been solved, but not those of full-length translocators assembled in a membrane-inserted translocon structure [78, 79]. Cryo-ET analysis revealed that the formation of the translocon leads to membrane bending and takes place in regions where the needle contacts the host cell, suggesting that a close association of bacteria and host is required for an efficient translocation [58, 80]. Moreover, the needle tip was shown to function as a guide for translocon formation [81]. The translocon diameter varies between approx. 13.5–60 nm, depending on the conditions in which was determined (i.e. *in vivo*/*in vitro*) [82].

### Type III secretion-independent assembly

For a successful infection, in competition with the host’s immune defence and the commensal microbiota, a timely and effective assembly of the intricate injectisome complex is essential.

Assembly of the injectisome can be divided into two parts: T3SS-independent and -dependent assembly. The first one utilizes the bacterial housekeeping protein secretion and membrane protein integration machinery (e.g. Sec, Bam, Lol) for building up the minimal secretion complex (export apparatus, base rings), which is complemented by the cytoplasmic components and then finished in a T3SS-dependent way.

Once the bacteria sense the right environmental signals, which vary among bacterial species, expression of the injectisome components is triggered. For example, in *Yersinia *, the host temperature of 37 °C activates the T3SS expression, whereas osmolarity, pH, and oxygen availability in the host intestinal environment serve as a trigger in *Salmonella* [83, 84]. Expression of the components leads to assembly of the injectisome complex to occur in about 30 min [19, 85]. Injectisome assembly begins with the formation of the core export apparatus in the IM [86] and with the formation of the secretin complex in the OM [87].

The core export apparatus assembles from a pseudo-hexameric SctR_5_T_1_ subcomplex through recruitment of four SctS followed by one SctU (Fig. 2a) [5–8]. During the assembly steps, inter- and intramolecular salt-bridges formed by highly conserved charged residues in the hydrophobic domains of the subunits play a crucial role in stabilization of the unique helical structure of the core export apparatus [13]. The SctV nonamer assembles around the core export apparatus which may support the displacement of the SctR_5_S_4_T_1_U_1_ complex from the IM [5, 9]. Surprisingly, SctV did not prove to be required for assembly of the base in *S*. Typhimurium T3SS-1 even though it is essential for secretion [86]. Instead, the core export apparatus was shown to nucleate assembly of the base, possibly by scaffolding SctJ oligomerization. In the absence of the core export apparatus, the base assembles inefficiently into a structure of wrong SctC (15 instead of 15.5) and SctDJ (23 instead of 24) stoichiometry [9, 34], highlighting the critical importance of the core export apparatus for base assembly. For SctD assembly, two models were proposed: in *Yersinia *, SctD assembles onto the pre-formed secretin [87], after which the export apparatus-SctJ complex and the secretin-SctD complex are thought to integrate. This model was termed the outside-in assembly model. Data from *Salmonella* T3SS-1 on the other hand suggest that SctD assembles onto or together with SctJ, after which the entire IM complex docks onto the OM secretin (inside-out model) [25, 88]. Both models have their challenges. For the outside-in model to work it is necessary that the SctD ring does not close completely as the export apparatus-SctJ complex must be able to integrate in the two-dimensional confinement of the IM. In the inside-out model it is in principle conceivable that the cytoplasmic components are recruited in the absence of the secretin, leading to the (premature) onset of secretion. Thus, checks and balances must exist to ensure the proper timing of the assembly and secretion processes that await elucidation.

A small lipoprotein called pilotin is important for assembly of the SctC secretin (Fig. 2a). The pilotin is anchored to the inner leaflet of the OM by a lipid anchor. It interacts with SctC monomers at the S domain, and targets them to the OM [31]. SctC then oligomerizes into a double-walled β-barrel [29]. In some pathogens including *Yersinia *, the pilotin is also implicated in oligomerization of SctC [32]. While in most studies, the secretin was shown to assemble independently of other T3SS components, except the pilotin, the formation of an SDS-resistant secretin oligomer depended on SctD in EPEC [89].

The cytoplasmic components were shown to exhibit a dynamic nature with cycles of assembly and disassembly [39, 44] (Fig. 2b). Since the cytoplasmic components are soluble, their assembly is not confined in a two-dimensional space and only limited by the affinity of the individual components to each other. Pod substructures may pre-form in the cytoplasm and play a role in targeting of substrates in a hierarchical fashion, for which reason the term ‘sorting platform’ was coined. The major integration point of the base and the cytoplasmic components is the SctK-SctD interaction [40]. It is unlikely, though, that SctK is recruited independently to SctD as the SctD ring possesses 24 potential binding sites while the cytoplasmic components feature a sixfold symmetry. This notion is supported by the observation that SctK shows a strongly diminished binding to SctD in the absence of SctQ when observed by fluorescence microscopy in *Yersinia* or cryo-ET in *Salmonella* [35, 39]. Interestingly, *in vivo* photocrosslinking showed an interaction of SctK with SctD in the absence of SctQ in *Salmonella *, even though its extent was reduced [37]. It is conceivable that in this case the binding of SctK to one of the 24 sites on SctD is possible albeit with an incorrect overall symmetry. In *Salmonella *, SctD organizes in six patches of four SctD N-terminal domains each, suggesting conformational changes to accommodate the hexameric symmetry of the cytoplasmic components [35]. However, this was not seen in *Shigella *’s T3SS [90], so the interaction between SctD and SctK may vary from system to system. Overall, it is conceivable that the hexameric ATPase enforces the symmetry of the pods. Pre-formed complexes of the pods (SctQK), the spoke (SctL) and one subunit of the ATPase (SctN) dock onto SctD and are forced by SctN-SctN-interactions into the hexameric symmetry. Alternatively, the cytoplasmic components pre-form the complete hexameric complex before docking onto SctD altogether. *In vitro* data point at the first model [45], which would also accommodate the observation of the dynamic exchange of substructures of the cytoplasmic components more easily. The last component to assemble is possibly SctO, which integrates into the pore of the hexameric SctN and protrudes towards and connects to SctV.

After assembly of the cytoplasmic components, the T3SS is thought to be secretion competent.

### Type III secretion-dependent assembly

Assembly of the injectisome is finalized by T3S-dependent processes that build up the needle filament and its associated structures. These processes require the targeting and secretion of proteins, i.e. substrates, through the otherwise assembled T3S apparatus (see *Targeting and transport across the injectisome*). Type III secretion-dependent assembly begins with the formation of the needle of the injectisome, followed by the insertion of translocators at the host membrane, which culminates in the secretion of effectors (Fig. 2c, d and e). Depending on the time of secretion, substrates of the T3SS can be grouped as: i) early substrates (components required for assembly of the needle filament); ii) intermediate substrates (needle tip/translocators); and iii) late substrates (effectors). The sequential secretion of the different substrates requires precise orchestration and distinct substrate specificity switches.

There are three early substrates common to all T3SS, the needle adapter SctI, the needle filament protein SctF and the needle length regulator SctP [88, 91]. In the *Salmonella* T3SS-1, a fourth early substrate, OrgC, has been reported to aid in polymerization of the needle filament at the distal end [92]. Whether there is a temporal regulation of secretion between the early substrates is not clear. It is clear though, that six subunits of the needle adapter SctI need to assemble onto the core export apparatus before assembly of the needle filament can begin, even though it was observed that SctI mutants with defects in needle assembly could be suppressed by overexpression of SctF, although inefficiently [93]. This suggests that SctI is not essential but facilitates the efficient anchoring of the needle filament to the core export apparatus, functioning as a flexible adapter protein. Interestingly, assembly of the SctI does not require prior assembly of SctC [55] and needle polymerization is still possible without SctC [25]. It has been postulated that secretion of SctI prior to secretin assembly activates a peptidoglycan-lytic enzyme in the periplasm of *Salmonella* in order to create space for subsequent assembly of the secretin [94]. However, it is unclear in this model how the secretin could assemble around a growing needle or how secretin and needle filament assembly are otherwise coordinated.

SctF polymerizes at the distal end of the needle [95] after travelling through the already assembled needle conduit (Fig. 2c). It is thought that the growing needle opens the secretin gate and then resumes in the extracellular space [29]. A variety of models of needle length control have been proposed over time [96]. It is now believed that the termination of needle elongation is regulated by the needle length regulator SctP and recent cryo-EM structure of the needle [30] suggests that it is independent of SctI. Deletion of SctP leads to extremely long needles and the oversecretion of SctI and SctF [18, 97–101]. How exactly SctP controls needle length has been debated for a long time [96, 102]. The most widely accepted model based on injectisome and flagella data states that SctP regulates needle length as an intermittently secreted infrequent ruler triggering the stop of needle elongation once the needle length exceeds the maximum extension of the unfolded SctP inside the needle conduit [59, 103]. SctP’s folded C-terminal domain is thought to trigger the substrate specificity switching to the secretion of intermediate substrates by interaction with the autocleaved C-terminal domain of SctU [99]. This interaction may induce a conformational change in SctU that disallows further secretion of early substrates and facilitates the binding of the gatekeeper SctW onto the cytoplasmic ring of SctV, which is postulated to be a prerequisite for secretion of intermediate substrates [17–19].

Upon substrate switching SctA (tip) is secreted and forms a pentameric ring whose subunits interact with SctF subunits in the same way as SctF subunits interact with each other [62]. This is in contrast with the previous model whereby the needle filament was thought to be topped by a tetrameric SctA together with an SctB (translocator) monomer [104]. Moreover, the insertion of SctA subunits at the top of the needle filament was shown to induce conformational changes in SctF [62]. Upon host cell contact, the needle tip may guide translocon formation [81]. The understanding of the mechanism of translocon assembly in the host membrane falls still short, due to a lack of high-resolution structures, but it seems to vary between different pathogens. After translocon insertion, assembly of the complete T3SS is finished and injection of effector proteins into the host cell can take place.

### Targeting and transport across the injectisome

For injection into host cells, T3SS substrates must be targeted to the injectisome, unfolded, and transported across it.

An essential requirement for T3S is the presence of a non-cleavable N-terminal signal within the first 20 amino acid residues [105–107]. This signal is necessary and sufficient for T3S and is universal, meaning the T3SSs of different bacteria can recognize it [108–111], however, there is no clear consensus sequence. The signal region is enriched in serine, threonine, isoleucine and proline, and is unstructured. Bioinformatic tools allow more accurate predictions of the signal [112, 113]. Exceptions, where T3S signals can be located in the middle of [100] and at both N- and C-terminus of the substrates have also been reported [114–116]. Also a potential relevance of mRNA signals has been discussed [107, 117].

In addition to the presence of the N-terminal signal sequence, many substrates also contain a chaperone binding domain (CBD) located downstream of the T3S signal that serves as a binding site for cognate T3SS chaperones [118]. T3SS chaperones have been shown to play different roles such as maintaining substrates unfolded [119], avoiding protein degradation [120], preventing mistargeting of transmembrane substrates [121], prevent self-aggregation [122] and facilitating the hierarchy of secretion [40, 123]. However, their functions seem specific for each substrate as not each chaperone performs all the functions. Moreover, not each T3SS substrate requires cognate chaperones for secretion [124].

Chaperones are classified depending on their substrate specificity. Class I chaperones bind to T3SS effectors. These are acidic proteins that share a similar 3D structure with αβα sandwich fold. They form homodimers. CDBs of effectors wrap around the chaperone dimers and prevent complete folding of the effector [125]. Class IA chaperones exclusively bind one effector whereas class IB chaperones bind multiple effector proteins. Class II chaperones are encoded in close proximity to and bind to the CBD and TMD of intermediate substrates such as translocators. They are also structurally similar and are composed of α-helices and tetratricopeptide repeat (TPR) domains [125]. A concave face of the chaperones binds the CDB, which contains a specific motif, and the convex back face binds the TMD [78]. They have been suggested to form dimers in solution, but the biological relevance of dimerization is unclear. Class III chaperones are structurally similar α-helical chaperones that bind flagellin and needle subunits (SctF), to the C-termini of their substrates [126–129]. Class IV chaperones are a group structurally different from the other classes, comprising the chaperone of the EPEC filament [130]. Class V chaperones also bind needle subunits and mediate contact of SctF with the T3SS apparatus [131] and regulate the levels of class III chaperone to translocate early substrates.

The presence of a T3S signal sequence and of a CBD may not be sufficient for the successful secretion of a protein through T3SS. On the contrary, several studies have shown that so far not all proteins are compatible with T3S. They probably cannot be unfolded sufficiently and thus obstruct the secretion channel [132–134], meaning that the mechanical stability of a protein can inform its compatibility with T3S [135].

The chain of events that occur from the expression of a substrate protein to its secretion by the injectisome are still elusive on the molecular level. Effector proteins seem to be immobilized in the cytosol, whether the needle complex and cytoplasmic components are present or not [136]. Moreover, the cytosolic components SctKQL appear to bind chaperones with different affinities, thus influencing effector export [40]. Dynamic assembly and disassembly of these components and their cycling between the cytoplasm and the injectisome were proposed to increase the probability of encountering a chaperone-substrate pair. However, cycles of assembly and disassembly were reported to have a minimum half-time of 1 min [44], while the rate of substrate secretion was shown to be in the range of 7–60 effectors per second [137]. It is difficult to match these different figures, questioning the relevance of this targeting hypothesis. Moreover, it was observed that chaperone-substrate pairs were capable of binding SctN and SctV [24, 50, 54, 138, 139]. It is not understood whether these interactions required a previous interaction with the SctKQL complex (multi-step targeting) or occur directly (one-step targeting).

Secretion of intermediate substrates depends also on the so-called gatekeeper SctW (Fig. 2d) that was shown to bind to the cytoplasmic ring of SctV [140]. SctW may act as a specific adapter for class II chaperone-substrate complexes to ensure timely secretion of intermediate substrates [138–142]. After host cell sensing and T3S activation, SctW dissociates from SctV and is secreted or degraded, so that the system switches to secretion of late substrates (Fig. 2d). Accordingly, in the absence of SctW late substrates are oversecreted [140, 143, 144]. Moreover, SctW expression was shown to be regulated by temperature through an upstream RNA thermometer [145]. Additionally, interaction of SctW with the ATPase was suggested to play a role in different binding affinities of early and intermediate substrates [146]. The hierarchy of secretion of different effectors may be regulated, e.g. by the regulation of the timing and strength of substrate expression and by the binding affinities between chaperones, substrates and injectisome components (Fig. 2e).

The secretion process itself is energized by the utilization of the proton motive force by the transmembrane domains of SctV, as well as by the hydrolysis of ATP by SctN [147–149]. Since secretion was also observed independently of ATPase activity, it seems that the main driver for secretion is the proton motive force, possibly leveraged by the ATPase [150, 151]. The underlying molecular mechanism of secretion is, however, not known to date. The actual crossing of the secretion conduit may also be driven by diffusion as it was proposed for bacterial flagella [152] and by folding of the substrate proteins when they emerge from the secretion conduit.

## Outlook

In recent years, the architecture of T3SS has been unveiled using different structural biology techniques such as cryo-ET, cryo-EM, and X-ray crystallography. The integrated picture obtained from all this structural information has led to a much improved understanding of the structure of the injectisome and to the generation of solid hypotheses on its function. However, some pieces are still missing, particularly transmembrane segments/regions and disordered or dynamic regions. The machine learning-based structure prediction tool Alphafold2 will certainly help to fill these gaps [153, 154]. Despite its only short time of availability, it has already been used to predict structures of needle tips and needle tip chimaeras [81], supported the solution of crystal structures by molecular replacement using Alphafold2 prediction [139] and helped to generate an integrated model of the cytoplasmic components of the *Salmonella* T3SS-1, which was then validated by *in vivo* photocrosslinking [37]. Beyond AlphaFold, molecular dynamics simulations may become increasingly useful to predict the movements of these large machines and thus infer function more accurately.
